# Microglial activation in the motor cortex mediated NLRP3-related neuroinflammation and neuronal damage following spinal cord injury

**DOI:** 10.3389/fncel.2022.956079

**Published:** 2022-10-20

**Authors:** Xvlei Hu, Yifan Zhang, Lei Wang, Jiangwei Ding, Mei Li, Hailiang Li, Liang Wu, Zhong Zeng, Hechun Xia

**Affiliations:** ^1^Department of Neurosurgery, Shanxi Provincial People's Hospital, Taiyuan, China; ^2^School of Clinical Medicine, Ningxia Medical University, Yinchuan, China; ^3^Ningxia Key Laboratory of Craniocerebral Diseases, Ningxia Medical University, Yinchuan, China; ^4^Ningxia Human Stem Cell Research Institute, General Hospital of Ningxia Medical University, Yinchuan, China; ^5^Department of Neurosurgery, Henan Provincial People's Hospital, Zhengzhou, China; ^6^Department of Neurosurgery, General Hospital of Ningxia Medical University, Yinchuan, China

**Keywords:** spinal cord injury, motor cortex, neuroinflammation, microglia, NLRP3 inflammasome

## Abstract

Spinal cord injury (SCI) is a traumatic event that can lead to neurodegeneration. Neuronal damage in the primary motor cortex (M1) can hinder motor function recovery after SCI. However, the exact mechanisms involved in neuronal damage after SCI remain incompletely understood. In this study, we found that microglia were activated in M1 after SCI, which triggered Nod-like receptor protein 3 (NLRP3) related chronic neuroinflammation and neuronal damage *in vivo*. Meanwhile, treatment with the microglia inhibitor minocycline reduced inflammation-induced neuronal damage in M1, protected the integrity of the motor conduction pathway, and promoted motor function recovery. Furthermore, we simulated chronic inflammation in M1 after SCI by culturing the primary neurons in primary microglia-conditioned medium, and observed that the injury to the primary neurons also occurred *in vitro*; however, as observed *in vivo*, these effects could be mitigated by minocycline treatment. Our results indicated that microglial activation in M1 mediates NLRP3-related neuroinflammation and causes the injury to M1 neurons, thereby impairing the integrity of the motor conduction pathway and inhibiting motor function recovery. These findings might contribute to the identification of novel therapeutic strategies for SCI.

## Introduction

Spinal cord injury (SCI) can lead to serious neurological impairment that often results in severe disability (Zipser et al., 2022). Motor function represents a basic physiological function in most animals and is thus critical to quality of life. Mechanical trauma in the spinal cord may lead to a partial or complete rupture of nerve fibers, resulting in complete or incomplete SCI. In complete SCI, motor function governed by the spinal cord segment below the injury site is completely lost. High cervical SCI often leads to quadriplegia, which is defined as the loss of lower limb motor function and a complete or partial loss of upper limb motor function. Meanwhile, lower limb paralysis resulting from low-plane SCI can affect overall flexibility and motor function coordination, and affected patients can lose their abilities to fully perform routine manual tasks, such as self-catheterization, personal hygiene, or eating (Fonseca et al., 2019).

The pathophysiological progression of SCI can be divided into primary and secondary injury. Primary injury refers to the injury induced by external mechanical forces. Secondary injury often leads to invasive degeneration of the spinal cord tissue, characterized by the apoptosis of neurons and glial cells (especially oligodendrocytes), axon retraction, inflammatory cell infiltration, the formation of glial scars, biochemical changes in excitatory amino acids, and the demyelination of neurons and their subsequent exposure to myelin-related inhibitory molecules, among other consequences (Fitch and Silver, 2008; Eli et al., 2021). In addition, SCI can also cause progressive neurodegeneration in the brain (Jure and Labombarda, 2017; Ziegler et al., 2018; Li et al., 2020a). Clinical studies have shown that SCI results in a reduction in volume, atrophy, and functional reorganization in the motor cortex (Wrigley et al., 2009; Freund et al., 2011, 2012; Hou et al., 2014). Meanwhile, in non-human primates, SCI results in reduced motor cortex activation, enhanced bilateral motor cortex interaction, and greater functional connectivity between the motor cortex and other brain regions (Chao et al., 2019; Suzuki et al., 2020). Studies on rodents have demonstrated that SCI leads to the loss of motor cortex neurons, through apoptosis or otherwise, and the activation of microglia (Hains et al., 2003; Li et al., 2020b). The cortical atrophy and functional reorganization found in clinical and primate studies are consistent with the damage or loss of cortical neurons observed in rodents. Interestingly, our recent studies found that metabolic activity is altered in the motor cortex after SCI (Wu et al., 2020; Zhang et al., 2021a).

At present, motor function recovery after SCI remains limited despite the development of promising therapeutic methods, such as cell transplantation, selective use of pharmacological agents, early surgical intervention-based bioengineering, and the use of methylprednisolone (Khorasanizadeh et al., 2019). The limited recovery of motor function is related not only to the complex biology of local tissues, but also to the neglect of brain changes after SCI (Isa, 2017). The brain is at the core of body movement management, and changes in the motor cortex may impede motor function recovery (Xie et al., 2021). These observations highlight the need for a comprehensive understanding of the biological mechanisms underlying neuronal injury in this brain region.

Microglia are the resident macrophages of the brain parenchyma and represent approximately 10% of all brain cells (Yirmiya et al., 2015; Bennett et al., 2018). They play an important role in brain development, the maintenance of normal brain structure, and the regulation of brain function. Microglia are activated under pathological conditions (such as infection, injury, and neurodegeneration) to coordinate and undertake neuroinflammatory and toxic responses. The Nod-like receptor protein 3 (NLRP3) inflammasome is an oligomeric complex comprising NLRP3, apoptosis-associated speck-like protein (ASC), and caspase-1 (de Rivero Vaccari et al., 2016). In the central nervous system, the NLRP3 inflammasome is expressed in microglia, and has been implicated in the pathology of neurodegenerative disorders, such as Alzheimer's disease, Parkinson's disease, traumatic brain injury, and depression (Pan et al., 2014; O'Brien et al., 2020; Holbrook et al., 2021). However, it is unclear whether microglial activation in M1 after SCI induces NLRP3 inflammasome-related chronic neuroinflammation and, consequently, neuronal damage.

In this study, we investigated neuroinflammation in M1 after SCI and sought to identify the associated biological mechanism both *in vivo*, using a rat model of SCI, and *in vitro*, employing rat primary cells. The aim of this study was to identify the novel targets that could aid in maximizing motor function recovery following SCI.

## Materials and methods

### Animals and groups

**Figure 1:**
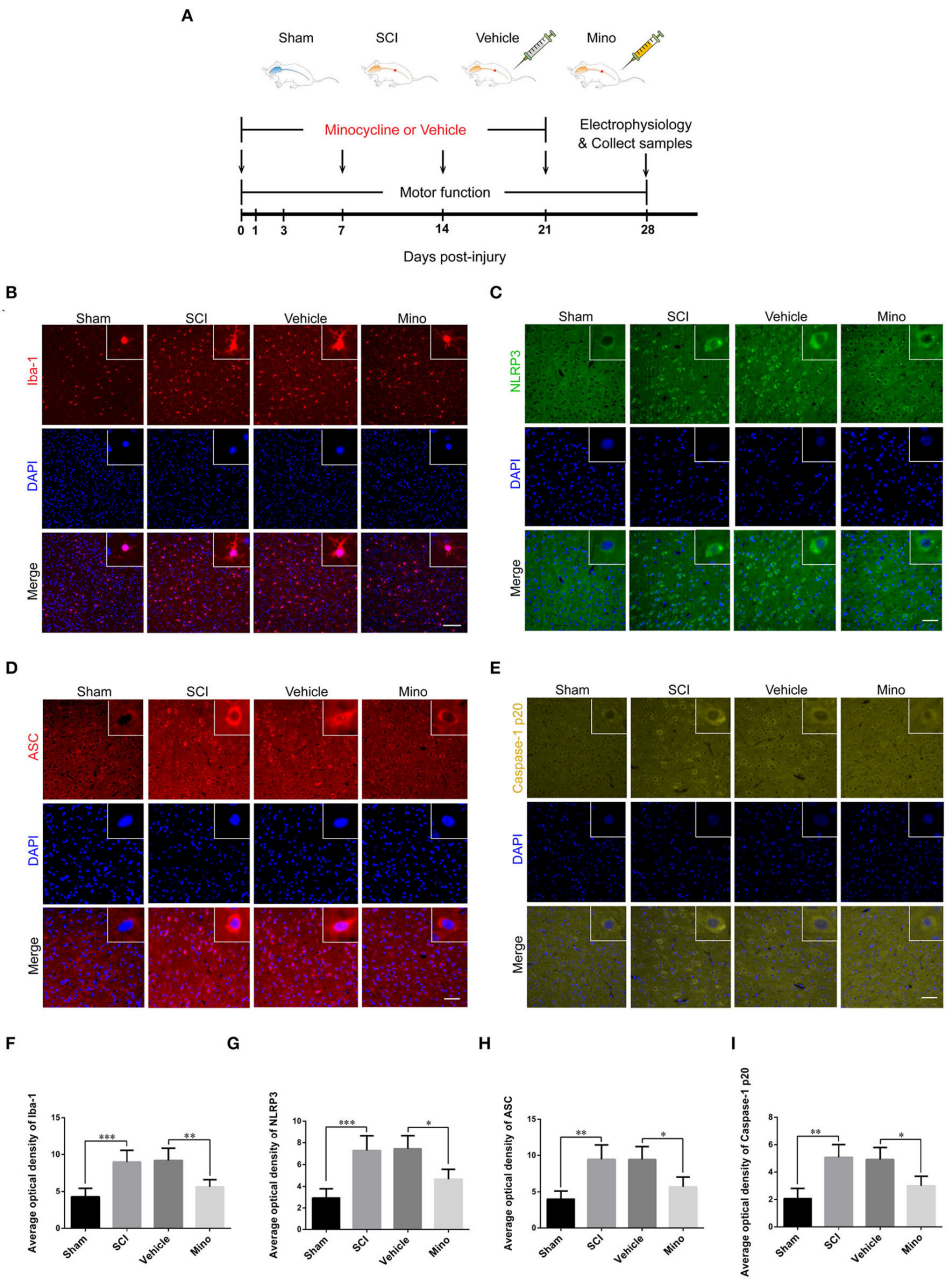
Microglial activation in M1 mediated Nod-like receptor protein 3 (NLRP3-) related neuroinflammation following spinal cord injury (SCI), which was inhibited by minocycline therapy. **(A)** Schematic diagram of the groups and study protocols employed *in vivo*. **(B–E)** Representative immunofluorescent images of Iba-1, NLRP3, ASC, and caspase-1 p20 in M1 of each group. Scale bars = 100 μm for Iba-1 and 50 μm for NLRP3, ASC, and caspase-1 p20. **(F–I)** The average optical density of Iba-1, NLRP3, ASC, and caspase-1 p20 in M1 of each group [data were presented as means ± standard deviation (SD), *n* ≥ 4, *p < 0.05, **p < 0.01, ***p < 0.001].

Adult female Sprague–Dawley (SD) rats weighing 250–300 g were obtained from the Experimental Animal Center of Ningxia Medical University. All animal experiments were approved by the Animal Research Ethics Committee of Ningxia Medical University. Animals were kept in a specific pathogen-free environment under a 12-h/12-h light/dark cycle with free access to food and water. After 1 week of adaptation to the environment, these rats were randomly assigned to four groups: Sham group, SCI group, Vehicle group, and Mino group (minocycline, microglia inhibitor). Sham group, in which animals received only laminectomy without SCI; SCI group, in which rats underwent laminectomy and spinal cord contusion; Mino group, in which SCI rats received minocycline (45 mg/kg, diluted in sterilized water, Yuanye Bio-Technology, China) intraperitoneally weekly (Zhang G. et al., 2019; Feng et al., 2021); and Vehicle group, in which SCI rats received the corresponding dose of sterilized water (see Figure 1A).

### SCI model

The rat model of SCI was generated, as previously described (Wu et al., 2014; Chang et al., 2021), with some modifications. Briefly, rats were anesthetized with 1% pentobarbital (1 g/100 ml) and hair was removed from the operation area. Once the T9–T10 segments were located, the skin and fascia were cut along the dorsal midline and the skin was pulled open with a hook to reveal the back muscles. The bilateral paraspinal muscles were sequentially separated to expose the T8–T11 vertebrae, following which the lamina was carefully removed and the T9–T10 segments were fully exposed. The head of the percussion device was aligned with the exposed spinal cord, and a 10-g weight was dropped from a height of 25 cm to the tail end of the percussion device, causing spinal cord contusion by force conduction (Supplementary Figure S1). Hind limb twitching and involuntary tail swinging indicated successful modeling. Animals were well cared for after surgery, with special attention paid to the bladder and rectum.

### Primary microglia-conditioned medium and primary neuronal culture

Newborn SD rats were sacrificed by inhaling excess isoflurane. After removing the skull, the pia meninges and blood vessels were stripped in the precooled D-Hanks buffer. Cortical tissues were separated from the brain and rinsed again and cut into small pieces. Then, these tissues were incubated in Trypsin-EDTA digestion solution at 37°C for 15–20 min. Observation was done every 5 min until the tilted surface was transparent, 10% Dulbecco's modified Eagle medium (DMEM) was added to terminate the digestion. After centrifugation (1,000 RPM, 5 min), the supernatant was removed and the resuspended cells were filtered through a 70-μm filter, yielding a single-cell suspension.

#### Primary microglia-conditioned medium

Cells at an appropriate density were cultured in polylysine-coated flasks. After 24 h, cells were rinsed three times with preheated PBS to remove impurities. Fresh complete DMEM medium was added and subsequently renewed every 3 or 4 days. On day 10, microglia could be seen in the uppermost layer. The flask was then placed on a shaker at a constant temperature for 12 h. The microglia could be separated by taking advantage of the weak adhesion property. Further, the primary microglia were divided into four groups: control group: complete medium cultured for 12 h, fresh complete medium cultured for another 12 h, then another fresh medium cultured for 24 h. Mino group: complete culture medium containing the microglia inhibitor minocycline cultured for 12 h, fresh complete medium cultured for another 12 h, then another fresh another medium cultured for 24 h. Lipopolysaccharide (LPS) + Nig group: complete medium cultured for 12 h, fresh complete medium containing LPS (1 μg/ml, Escherichia coli O111:B4) and nigericin (10 μM, added at 11 h) (Nam et al., 2018; Zeng et al., 2019) cultured for another 12 h, then another fresh medium cultured for 24 h. LPS+Nig+Mino group: complete culture medium containing the microglia inhibitor minocycline cultured for 12 h, complete fresh medium containing LPS (1 μg/ml) and nigericin (10 μM, added at 11 h) cultured for another 12 h, then another fresh medium cultured for 24 h (see **Figure 5A**).

#### Primary neuron culture in microglia-conditioned medium

As for the primary neurons, the single-cell suspension was cultured in neurobasal medium (Gibco, USA) with 2% B27, 1% L-glutamine, and 1% penicillin-streptomycin solution. Half of the medium was replaced every 3 or 4 days. On day 9, primary cortical neurons were cultured in microglia-conditioned medium (Control, Mino, LPS+Nig, and LPS+Nig+Mino groups) for 24 h (see **Figure 6A**).

### ELISA assay

Primary microglia-conditioned medium was harvested from each group, and the levels of IL-18 (EK0592), IL-1β (EK0393), TNF-α (EK0526), and IL-6 (EK0412) were evaluated using associated enzyme-linked immunosorbent assay (ELISA) kits (BOSTER, Wuhan, China), according to the manufacturer's instructions. The absorbance was measured at a wavelength of 450 nm.

### Cell viability assay

Cell viability was measured using a Cell Counting kit (TransDetect, Beijing, China), according to the manufacturer's protocol. Briefly, primary microglia were seeded in 96-well plates. After adhesion, different doses of minocycline (10, 25, 50, 75, and 100 μM) were added to the wells for 12 h. After treatment (minocycline or microglia-conditioned medium), complete medium containing 10% cholecystokinin (CCK) was added to the wells for 1 h. The absorbance was assessed at a wavelength of 450 nm.

### Flow cytometry

Primary neurons cultured in microglia-conditioned medium were centrifuged (4°C, 300 × g, 5 min) and washed two times with precooled PBS, and the cell density was adjusted to 1 × 10^6^ cells/ml with the addition of 400 μl of Annexin V binding solution. The cells were then incubated for 15 min with Annexin V–FITC staining solution (5 μl) and then for 5 min with propidium iodide (PI) staining solution (5 μl) at 4°C. Finally, the level of apoptosis was determined by flow cytometry.

### Western blot analysis

The rats were deeply anesthetized. The skull was decapitated and stripped off, then the brain could be integrally removed. The pial and blood vessels on the brain surface were removed on ice and placed into a precooled rat brain mold. According to the rat brain atlas, the excess brain tissue was discarded along the corona and a certain region of the M1 was retained. Total protein was extracted from a fresh M1 tissue using the Total Protein Extraction kit (KGP2100, KeyGEN BioTECH, China), and protein concentrations were determined using the BCA Protein Assay kit (KGPBCA, KeyGEN BioTECH, China). Equal amounts of protein were separated by sodium dodecyl sulfate-polyacrylamide gel electrophoresis (SDS-PAGE) and then transferred to 0.2-μm polyvinylidene difluoride (PVDF) membranes (10600021, Amersham, Germany). Then, 5% bovine serum albumin (BSA) was applied to block non-specific binding at room temperature for 2 h. Next, the membrane was incubated with the following primary antibodies at 4°C overnight: NLRP3 (bs-10021R, Bioss, China), ASC (bs-6741R, Bioss, China), caspase-1 p20 (bs-10442R, Bioss, China), IL-1β (bs-0812R, Bioss, China), IL-18 (bs-4988R, Bioss, China), TNF-α (bs-10802R, Bioss, China), IL-6 (DF-6087, Affinity, USA), Active Caspase3 (bsm-33199M, Bioss, China), and β-actin (66009-1-Ig, Proteintech, USA) (all 1:1000). After washing three times in 0.1% tris-buffered saline containing 5% Tween-20, membranes were incubated in appropriate secondary antibodies at room temperature for 2 h: Goat anti-Mouse (1:4000, 926-32210, LI-COR, USA) and Goat anti-Rabbit (1:4000, 926-32211, LI-COR, USA). Finally, these membranes were visualized in an Odyssey Infrared Imaging System (CLX-0796, Gene Company Limited, USA) and quantified with ImageJ software (National Institutes of Health).

### Nissl staining

Rats were deeply anesthetized and transcardially perfused with 0.9% saline (250 ml, room temperature) and 4% paraformaldehyde solution (500 ml, 4°C). Then, the brains were prepared into 5-μm-thick paraffin sections. After dewaxing and dehydration, the sections were incubated in Nissl staining solution (G1436, Solarbio, China) for 30 min at 50–60°C. Subsequently, these sections were washed with flowing water, dehydrated with 100% ethanol, and cleared with xylene. Finally, stained sections were imaged under a light microscope.

### Immunofluorescence

The protocol used to prepare the tissue sections was the same as Nissl staining. Then, the sections were boiled in sodium citrate antigen repair solution for 20 min, and immersed in 0.5% Triton X-100 for 30 min. Also, cultured microglia and neurons were harvested and fixed. The brain sections or cells were blocked in 5% BSA for 2 h at room temperature. Subsequently, these sections or cells were incubated with the following primary antibodies at 4°C overnight: Iba-1 (1:500 brain sections and 1:1000 cells, ab178846, Abcam, UK), NLRP3 (1:400 brain sections and 1:1000 cells, bs-10021R, Bioss, China), ASC (1:400 brain sections and 1:1000 cells, bs-6741R, Bioss, China), caspase-1 p20 (1:400 brain sections and 1:1000 cells, bs-10442R, Bioss, China), NeuN (1:500, ab177487, Abcam, UK), Active Caspase3 (1:400, bsm-33199M, Bioss, China), and MAP2 (1:200, GTX133109, GeneTex, USA). After washing three times in PBS containing 5% Tween-20, brain sections and cells were incubated in appropriate secondary antibodies at 4°C for 8 h: Goat Anti-Rabbit IgG FITC (1:100, SA00003-2, Proteintech, USA) and Goat Anti-Mouse IgG Alexa Fluor 647 (1:100, ab150115, Abcam, UK), after which cell nuclei were stained with DAPI. Finally, brain sections and cells were imaged under a multichannel fluorescence microscope (Leica, Wetzlar, Germany) and quantified with ImageJ software.

### Electrophysiological assay

Motor-evoked potentials (MEPs) were measured using electrophysiology (Redondo-Castro et al., 2016; Chen et al., 2020). Animals were anesthetized. A suspension silver sphere electrode as an anode was placed in the hindlimb representation of the motor cortex, just touching the dura without compression. The reference electrode as a cathode was placed under the skin between the ears. The recording electrode was inserted into the tibialis anterior muscle, and the grounding electrode was subcutaneously inserted. A single electrical pulse (10 mA, 0.1 ms, and 1 Hz) was used to stimulate the brain and record the amplitude of MEPs.

### Locomotion tests

Locomotor function was assessed using the Basso, Beattie, and Bresnahan (BBB) rating scores and the inclined plate test on days 1, 3, 7, 14, 21, and 28 after the operation (Basso et al., 1995; Gu et al., 2020). Rats were placed in an open field area or an inclined plane. The average BBB rating scale score and the maximum angle of the plane were assessed and recorded by two evaluators blinded to the experimental conditions.

### Statistical analysis

All data were analyzed in GraphPad Prism 6.01 (GraphPad Software, USA) and are presented as mean ± standard deviation (SD). One-way analysis of variance (ANOVA) with the Bonferroni test was applied for comparisons among multiple groups. A *p*-values < 0.05 were considered significant.

## Results

### Microglial activation in M1 mediated NLRP3-related neuroinflammation following SCI

**Figure 2:**
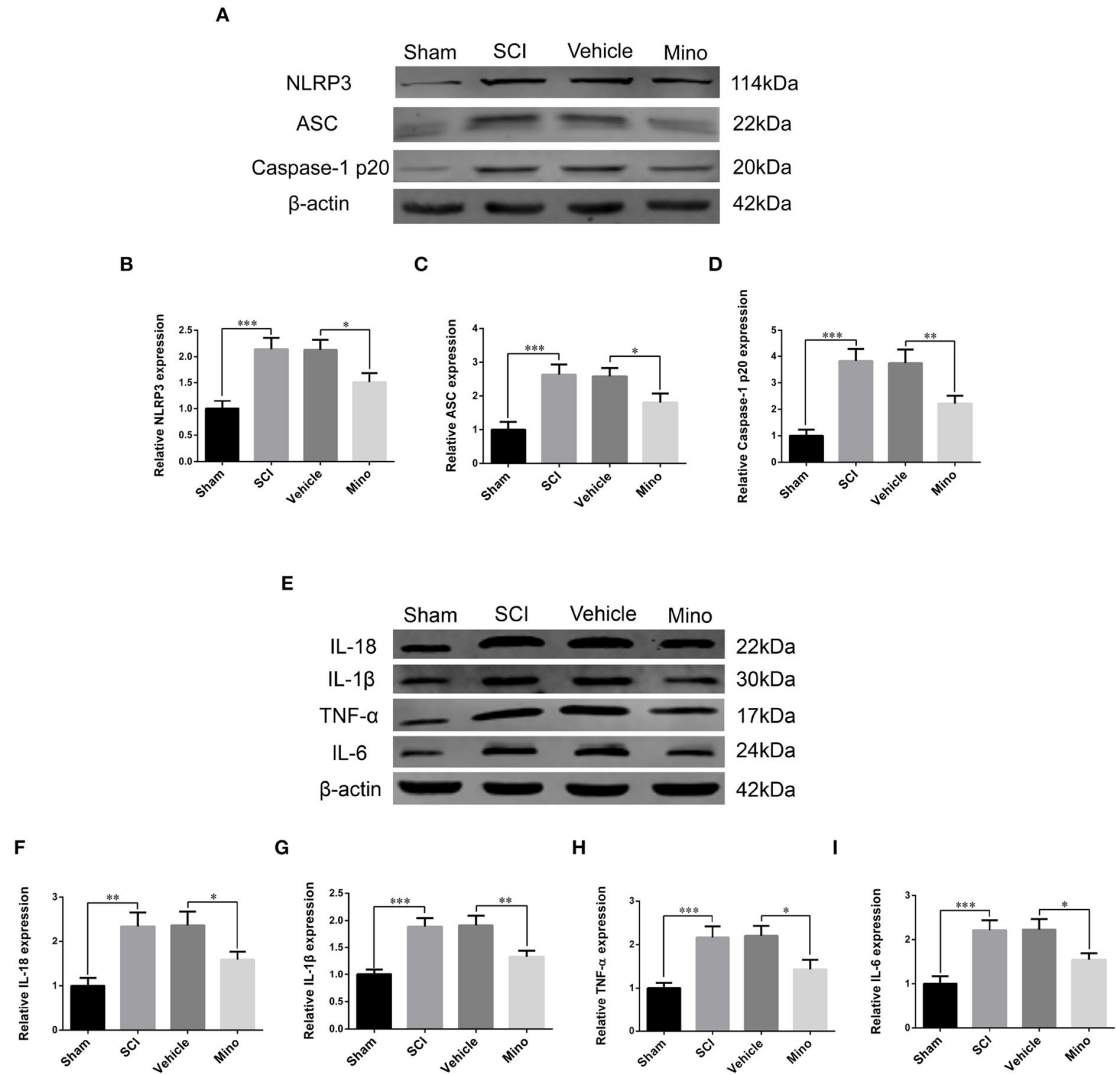
Western blot (WB) detected NLRP3-related neuroinflammation in M1. **(A)** Representative immunoblots of NLRP3, ASC, and caspase-1 p20 in M1 of each group. **(B–D)** Quantitative analyses of NLRP3, ASC, and caspase-1 p20 expression in M1 of each group. **(E)** Representative immunoblots of IL-18, IL-1β, TNF-α, and IL-6 in M1 of each group. **(F–I)** Quantitative analyses of IL-18, IL-1β, TNF-α, and IL-6 expression in M1 of each group (data were presented as means ± SD, *n* = 3, *p < 0.05, **p < 0.01, ***p < 0.001).

To assess the microglial activation status, we employed immunofluorescence staining to detect the expression of Iba-1, a microglial marker protein, and NLRP3, ASC, and caspase-1 p20 (caspase-1 subunit), components of the NLRP3 inflammasome (Figures 1B–E). Microglial activation was evident in M1 after SCI; however, this effect was blocked by minocycline treatment (an inhibitor of microglial activation) (Chen et al., 2018; Wang et al., 2018; Su et al., 2019). Meanwhile, the average optical density of NLRP3, ASC, and caspase-1 p20 was high after SCI, whereas the opposite was seen after minocycline treatment (Figures 1F–I). Similarly, western blot (WB) analysis revealed that the expression of NLRP3, ASC, and caspase-1 p20 was high after SCI, but this effect was blocked with minocycline treatment (Figures 2A–D). Given that increased NLRP3 inflammasome activation can lead to the maturation of IL-18 and IL-1β, which further promotes the secretion of TNF-α and IL-6, we next detected the expression of IL-18, IL-1β, TNF-α, and IL-6 in M1. As expected, the expression of all four proteins was increased following SCI, but returned to near-normal levels after minocycline treatment (Figures 2E–I). These findings suggested that microglial activation mediated NLRP3-related neuroinflammation in M1 following SCI.

### NLRP3-related neuroinflammation in M1 induced neuronal damage

**Figure 3:**
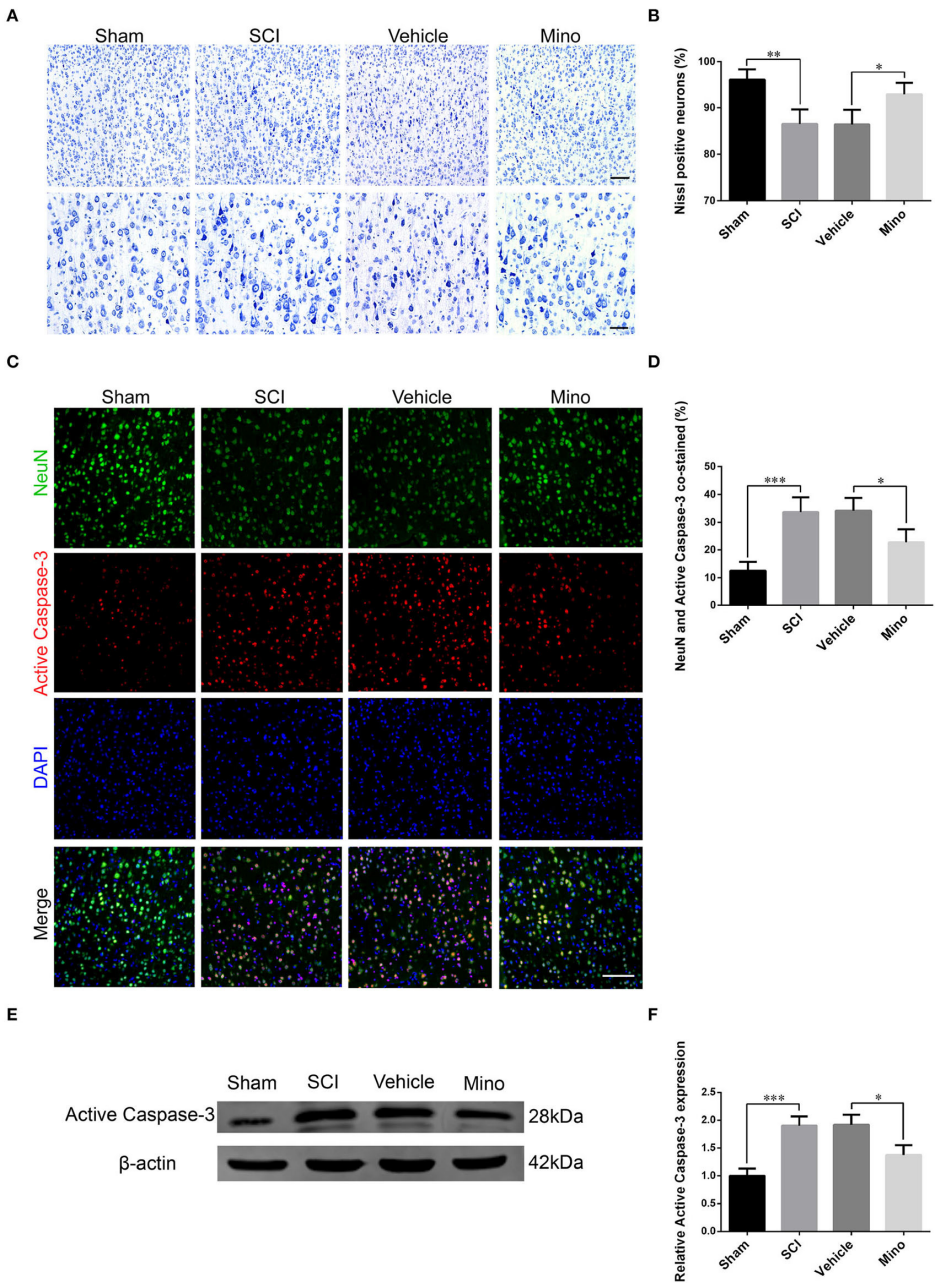
Neurons in M1 were damaged after SCI, an effect that was reversed by minocycline therapy. **(A)** Representative images of Nissl staining and **(B)** quantitative analysis of Nissl-positive neurons in M1 of each group. Scale bars = 100 (upper panels) and 50 μm (lower panels). **(C)** Representative immunofluorescence images and **(D)** quantitative analyses of NeuN and cleaved caspase-3 co-staining in M1 in each group. Scale bars = 100 μm. **(E)** Representative immunoblots and **(F)** quantitative analyses of cleaved caspase-3 expression in M1 of each group (data were presented as means ± SD, *n* = 4 for Nissl staining and immunofluorescent, *n* = 3 for WB, *p < 0.05, **p < 0.01, ***p < 0.001).

Damaged neurons often undergo apoptosis, and their ability to recover is limited. Determination is made whether an increase in inflammation in M1 caused an injury to neurons. We performed Nissl staining and evaluated the expression of cleaved (activated) caspase-3, a marker of apoptosis. The results showed that the number of Nissl-positive neurons was decreased, while that of NeuN/cleaved caspase-3 double-positive neurons was increased after SCI (Figures 3A–D). Similarly, cleaved caspase-3 protein levels were also increased after SCI (Figures 3E,F). However, both these effects were mitigated by minocycline treatment.

### Suppressing neuroinflammation in M1 improved the integrity of the motor conduction pathway and promoted locomotor function recovery after SCI

**Figure 4:**
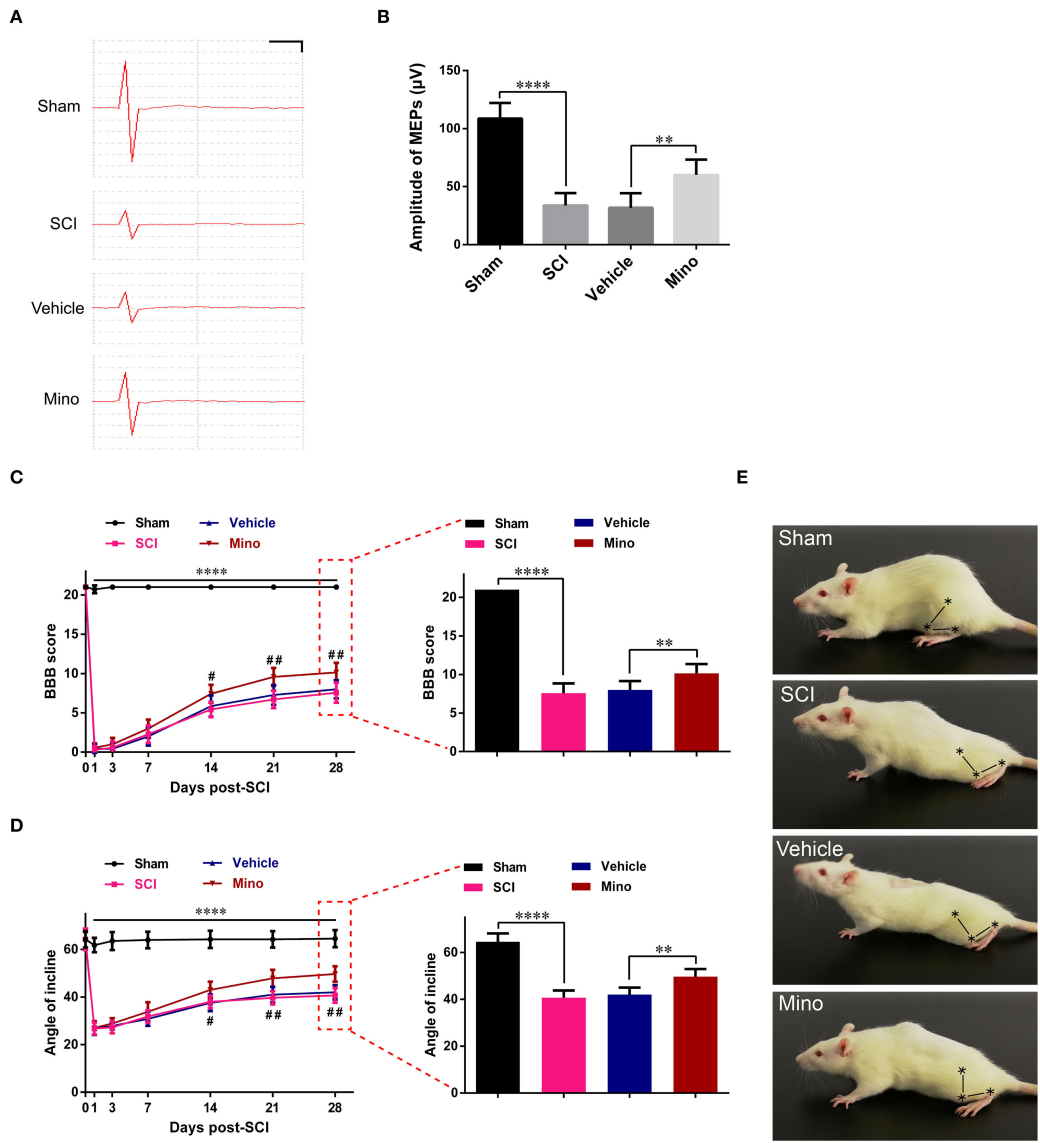
Suppressing neuroinflammation in M1 improved the integrity of the motor pathway and enhanced locomotor recovery after SCI. **(A)** Representative motor-evoked potentials (MEPs) and **(B)** quantitative analysis of the amplitudes of MEPs of each group (scale: 25 μV/1 ms). **(C)** The BBB scores and **(D)** angles of inclination of each group at different time points. **(E)** Representative motor function for each group. The symbols represent the hip, knee, and ankle joints (data were presented as means ± SD, *n* ≥ 6; ****p < 0.0001 Sham group vs. SCI group, ^#^*p* < 0.05, ^*##*^*p* < 0.01 Vehicle group vs. Mino group, for left panels; **p < 0.01, ****p < 0.0001).

Next, we investigated the functional integrity of the motor pathway using MEPs. The results showed that the amplitude of MEP was markedly reduced after SCI; however, this reduction was reversed following minocycline treatment (Figures 4A,B). Subsequently, the BBB rating scale and inclined plane test scores were determined as a readout of locomotor function. We found that locomotor function was severely curtailed after SCI, whereas the opposite was seen when minocycline was administered (Figures 4C–E). In total, our results suggested that suppressing neuroinflammation in M1 could improve the integrity of the motor pathways and enhance locomotor function recovery after SCI.

### NLRP3-related neuroinflammation induced primary cortical neuron damage

**Figure 5:**
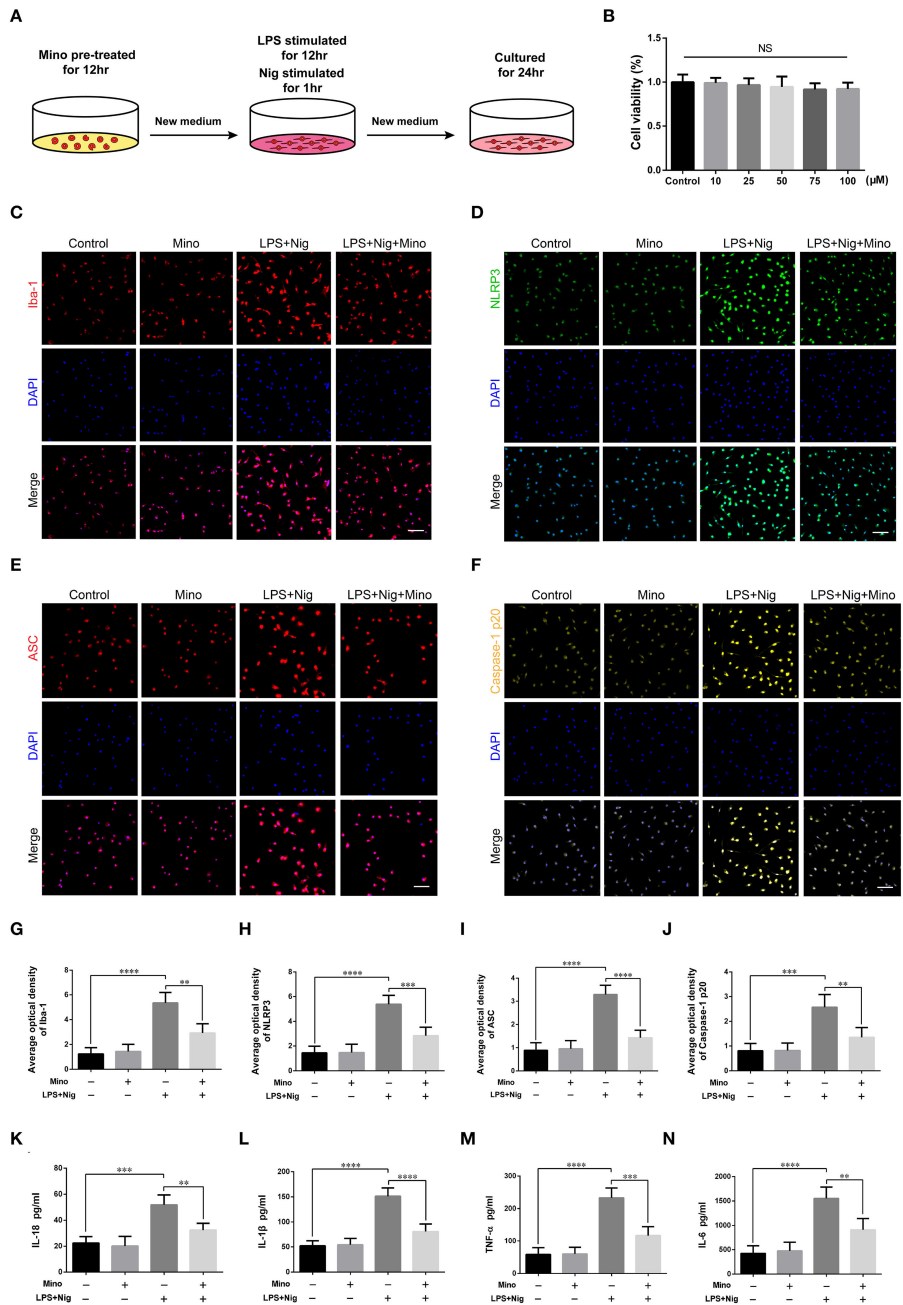
Lipopolysaccharide (LPS) and Nig induced activation of primary cortical microglia simulated NLRP3-related inflammatory environment *in vitro*. **(A)** Experimental protocols related to primary microglia. **(B)** The viability of primary microglia was assessed at different doses of minocycline. **(C–F)** Representative images of Iba-1, NLRP3, ASC, and caspase-1 p20 immunofluorescence in the primary microglia of each group. Scale bars = 50 μm. **(G–J)** The average optical densities of Iba-1, NLRP3, ASC, and caspase-1 p20. **(K–N)** Assessment of the levels of IL-18, IL-1β, TNF-α, and IL-6 secretion in primary microglia-conditioned medium using enzyme-linked immunosorbent assay (ELISA) [data are presented as means ± SD, *n* = 3 for cholecystokinin (CCK), *n* = 4 for immunofluorescence and ELISA assays; NS: *p* > 0.05, **p < 0.01, ***p < 0.001, ****p < 0.0001].

**Figure 6:**
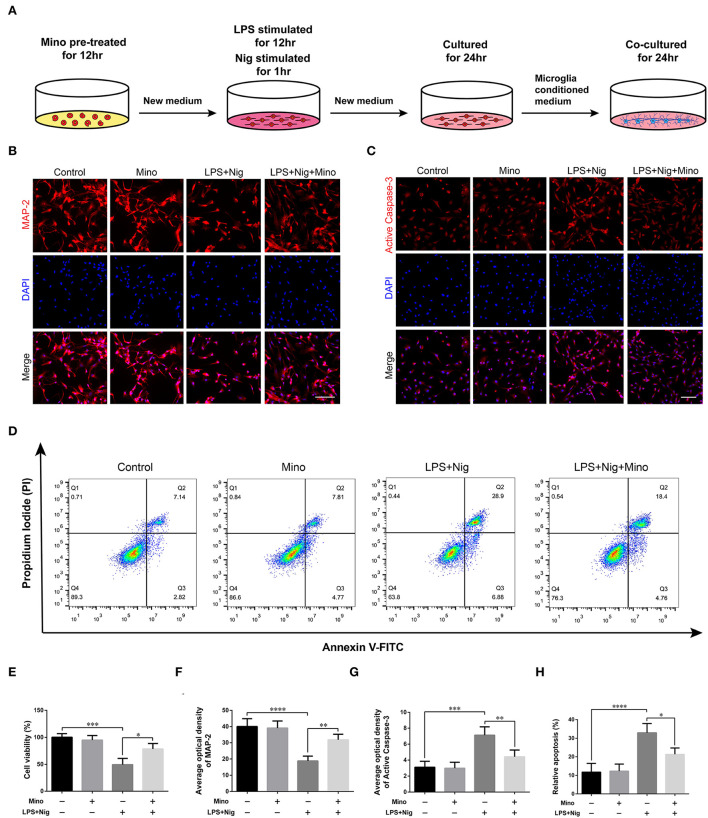
Dampening NLRP3-associated inflammatory environment alleviated the damage in primary cortical neurons cultured in microglia-conditioned medium. **(A)** Experimental protocols related to primary neurons. **(B,C)** Representative images of MAP2 and cleaved caspase-3 immunofluorescence in primary neurons cultured in microglia-conditioned medium of each group. Scale bars = 100 μm. **(D)** Representative images of cell apoptosis in primary neurons of each group as determined by flow cytometry. **(E)** The viability of primary neurons was measured using a CCK assay. **(F–H)** Quantitative analyses of the average optical densities of MAP2 and cleaved caspase-3, and apoptosis of each group (data are presented as means ± SD, *n* = 3 for the CCK assay, *n* = 4 for the immunofluorescence and flow cytometric assays; *p < 0.05, **p < 0.01, ***p < 0.001, ****p < 0.0001).

We successfully cultured primary cortical microglia and neurons with >95% positive cells of landmark protein (Supplementary Figures S2, S3). To determine the effect of minocycline on cell viability of primary microglia, a dose-response experiment was performed on microglia treated with minocycline for 12 h. Similar to previous studies (Lu et al., 2016; Piotrowska et al., 2017), cell viability was not changed in response to minocycline (10–100 μM) (Figure 5B). And, we chose 50 μM minocycline treatment for 12 h at subsequent experiments. Microglia were activated after LPS+Nig treatment, which were prevented by minocycline pretreatment. Meanwhile, the average optical density of NLRP3, ASC, and caspase-1 p20 was high after LPS+Nig treatment, and the average optical density of these three proteins was low after minocycline pretreatment (Figures 5C–J). The secretion of IL-18, IL-1β, TNF-α, and IL-6 was high after LPS+Nig treatment, which was also restrained after minocycline pretreatment (Figures 5K–N). Thus, we acquired microglia-conditioned medium. We further found that primary cortical neurons cultured in microglia-conditioned medium following LPS+Nig treatment experienced injury. In this group, cell viability and the average optical density of MAP2 were reduced, whereas the average optical density of cleaved caspase-3 and the levels of apoptosis were increased. Furthermore, negative effects associated with the activated microglia-conditioned medium were reversed with minocycline pretreatment (Figure 6). In total, the vitro results demonstrated that microglial activation mediated NLRP3-related neuroinflammation, resulting in damage to cortical neurons.

## Discussion

**Figure 7:**
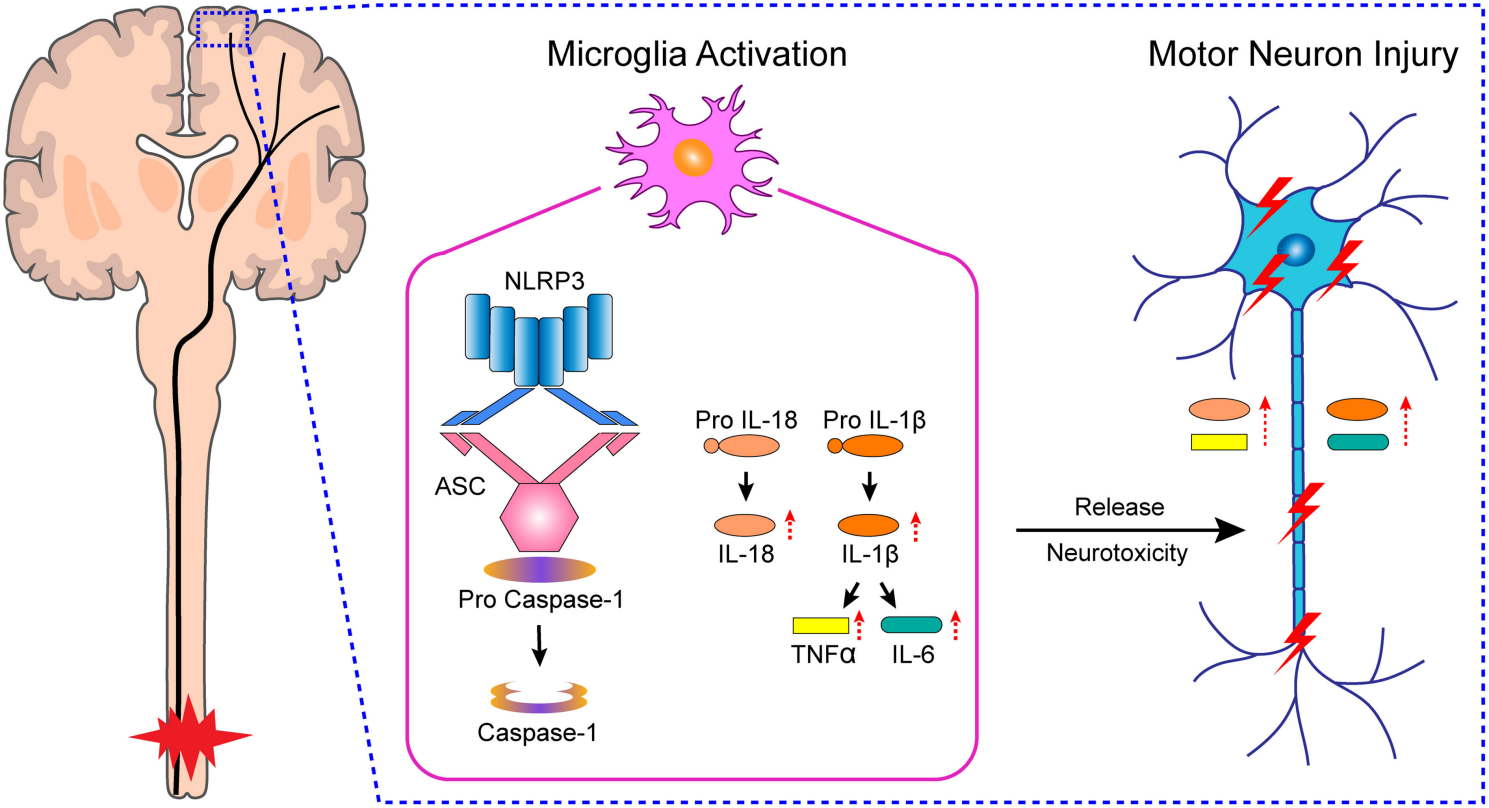
Hypothetical mechanism of neuronal damage in M1 of SCI rats.

In this study, we found that microglial activation mediated NLRP3-related neuroinflammation in the motor cortex after SCI, which resulted in the injury to motor neurons, affected the integrity of motor pathways, and delayed motor function recovery (Figure 7). Meanwhile, treatment with the microglial activation inhibitor minocycline attenuated the inflammatory environment, prevented neuronal damage, improved the integrity of motor pathways, and promoted motor function recovery. Furthermore, we cultured primary microglia derived from the rat M1 *in vitro*, and obtained microglia-conditioned medium containing excess inflammatory factors by treating the cells with drugs, which simulated the *in vivo* inflammatory environment. We found that cultured primary neurons exposed to microglia-conditioned medium also experienced extensive damage. However, pretreatment with minocycline reduced the levels of secreted inflammatory factors in microglia-conditioned medium and prevented the injury to primary neurons.

As an important part of the cerebral immune system, microglia with varied morphology and flexible mobility ensure real-time monitoring and a rapid response to maintain brain homeostasis (Dumas et al., 2021). Moreover, microglia can change their phenotypes according to environmental cues (Bernier et al., 2020), and are activated under neuroinflammatory conditions when they display a larger body and a greater number of branches and gain the ability to phagocytize cell debris and release cytokines. Studies have found that patients with SCI are often in a chronic inflammatory state, characterized by a 2–3-fold increase in the levels of circulating inflammatory markers compared with healthy people (Neefkes-Zonneveld et al., 2015). Similarly, in rats with SCI, the levels of blood inflammatory cytokines also remain high for extended periods (Maldonado-Bouchard et al., 2016). In addition to peripheral blood, chronic neuroinflammation is also prominently manifested in multiple brain regions (Wu et al., 2016; Xue et al., 2019). As for the spinal cord tissue, the accumulation of the blood–spinal cord barrier (BSCB) opening and inflammation were observed right after the injury, peaked at 7 days (Zhang L. et al., 2019; Zhang et al., 2021b). As for the brain, Wu et al. (2014) showed that microglia were activated in a wide area of the rat brain at 7 days and last for 10 weeks following SCI, with a reduced number of neurons in the cerebral cortex, thalamus, and hippocampus. Feng et al. (2021) showed that microglia were activated in the rat M1 in response to SCI; these microglia secreted a large amount of nitric oxide, and then regulated the expression of iron metabolism-related proteins, thus inducing ferroptosis in motor neurons. Our results are in agreement with these findings of the activation of microglia in M1 following SCI.

The innate immune response plays an important role in central nervous system injury. Pattern recognition receptors, such as Nod-like receptors, RIG-like receptors, and Toll-like receptors, recognize danger-associated molecular patterns, leading to the production of inflammatory cytokines, such as IL-1β, IL-18, TNF, and type I interferon, which subsequently activate the adaptive immune response (de Rivero Vaccari et al., 2014). The inflammasome is a Nod-like receptor-based multi-protein complex. In the central nervous system, the inflammasome is mainly associated with NLRP1, NLRP2, NLRP3, and AIM2. NLRP1 and AIM2 inflammasomes are found in neurons (Yuan et al., 2020), NLRP2 inflammasome in astrocytes (Minkiewicz et al., 2013), and the NLRP3 inflammasome in activated microglia (Shi et al., 2013); the NLRP3 inflammasome comprises NLRP3, ASC, and caspase-1. During the formation of NLRP3 inflammasomes, caspase-1 is converted from its proenzyme (inactive) form to its activated (cleaved) form, which induces the maturation and secretion of IL-1β and IL-18; the former further promotes the secretion of IL-6 and TNF-α (Alomar et al., 2016; Wang et al., 2021). Accordingly, we detected the levels of NLRP3, ASC, caspase-1, IL-1β, IL-18, IL-6, and TNF-α both *in vivo* and *in vitro*. We found that the expression of these factors was increased after SCI or LPS+Nig stimulation, an effect that was inhibited by minocycline treatment. We further assessed the damage to motor neurons in M1 using Nissl staining and the detection of apoptosis-related protein cleaved caspase-3. The Nissl body is a characteristic structure of neurons. When neurons are damaged, Nissl bodies disintegrate or even disappear. Here, we found that the number of Nissl-positive neurons in M1 was decreased, whereas the number of cells co-expressing NeuN and active caspase-3 in M1 was increased after SCI, indicative of increased levels of apoptosis. To further confirm this, we evaluated the expression of MAP2, a neurite marker, in primary neurons cultured in microglia-conditioned medium. MAP2 is stably expressed in neurons and can well represent their structural characteristics. MAP2 expression and the levels of apoptosis among the primary neurons, as determined by flow cytometry, were consistent with the *in vivo* findings.

Electrophysiology remains the gold standard in evaluating neurologic function. MEPs are frequently used to assess the integrity of the motor pathway and thereby judge the severity or the recovery of SCI. In this study, we found that the amplitude of MEPs was reduced and motor function recovery was limited after SCI in rats. However, minocycline treatment reversed these effects. It needs to be clarified whether the improvement of MEPs and motor function is due to the effect of minocycline on M1 inflammation. Local SCI is a traumatic event that often requires early treatment with large doses of drugs. In this study, however, the dosage and frequency of minocycline administration were designed based on its effects on neurodegeneration (Feng et al., 2021); consequently, the optimal treatment time was missed. This suggested that improvements observed in MEPs and motor function were mainly related to minocycline-mediated inhibition of neuro-inflammation in M1.

In summary, our findings suggest that microglial activation in M1 mediates NLRP3-related neuroinflammation and neuronal damage following SCI, which further affects the integrity of the motor pathway and hinders motor function recovery. Preventing the neurotoxicity of NLRP3 inflammasome on cortical motor neurons may be a promising therapeutic strategy to promote motor function recovery after SCI.

## Data availability statement

The original contributions presented in the study are included in the article/Supplementary material, further inquiries can be directed to the corresponding author/s.

## Ethics statement

The animal study was reviewed and approved by Animal Research Ethics Committee of Ningxia Medical University.

## Author contributions

XH and HX conceived and designed this study. XH, YZ, LWa, and ML performed the experiments. JD and HL analyzed the data. XH and ZZ prepared the figures. HX and LWu supervised this study. XH, YZ, and LWu wrote this article. All authors contributed to this article and approved the submitted version.

## Funding

This work was supported by the Key Research Projects of Ningxia Hui Autonomous Region, Grant Numbers (2018BCG01002 and 2020BEG03015).

## Conflict of interest

The authors declare that the research was conducted in the absence of any commercial or financial relationships that could be construed as a potential conflict of interest.

## Publisher's note

All claims expressed in this article are solely those of the authors and do not necessarily represent those of their affiliated organizations, or those of the publisher, the editors and the reviewers. Any product that may be evaluated in this article, or claim that may be made by its manufacturer, is not guaranteed or endorsed by the publisher.

## Supplementary material

Supplementary Figure S1 The establishment of the spinal cord injury (SCI) model in rats. **(A,B)** Skin preparation and disinfection. **(C–E)** Separation of the paravertebral muscles. **(F–K)** Dissection of the T9-T10 vertebral segments and induction of spinal cord contusion.

Supplementary Figure S2 Primary cultures of cortical neurons. **(A)** The number and morphology of primary neurons at different time points. Scale bars = 200 (upper panels) and 100 μm (lower panels). **(B)** Neuronal purity as determined by immunofluorescence. Scale bars = 100 μm.

Supplementary Figure S3 Primary cultures of cortical microglia. **(A)** The number and morphology of primary microglia at different time points. Scale bars = 100 (upper panels) and 50 μm (lower panels). **(B)** Microglial purity as determined by immunofluorescence. Scale bars = 50 μm.
