# Supplementary figures and images for: Microglial activation in the motor cortex mediated NLRP3-related neuroinflammation and neuronal damage following spinal cord injury
Source: Front Cell Neurosci. 2022 Oct 20;16:956079. doi: 10.3389/fncel.2022.956079 (PMC9630363; doi:10.3389/fncel.2022.956079)

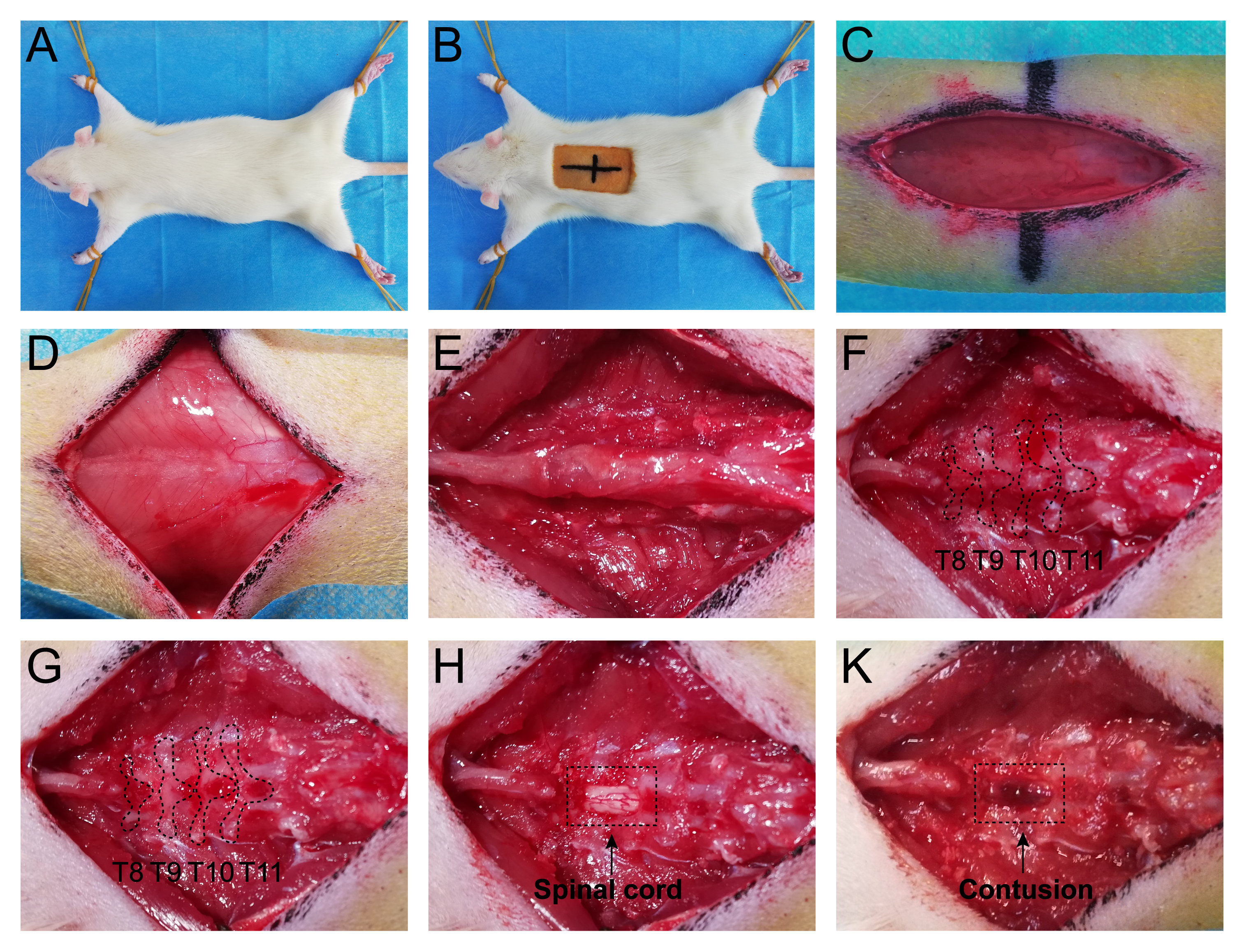

Supplement: Supplementary Figure S1 — The establishment of the spinal cord injury (SCI) model in rats. (A,B) Skin preparation and disinfection. (C–E) Separation of the paravertebral muscles. (F–K) Dissection of the T9-T10 vertebral segments and induction of spinal cord contusion. [file Image_1.TIF]

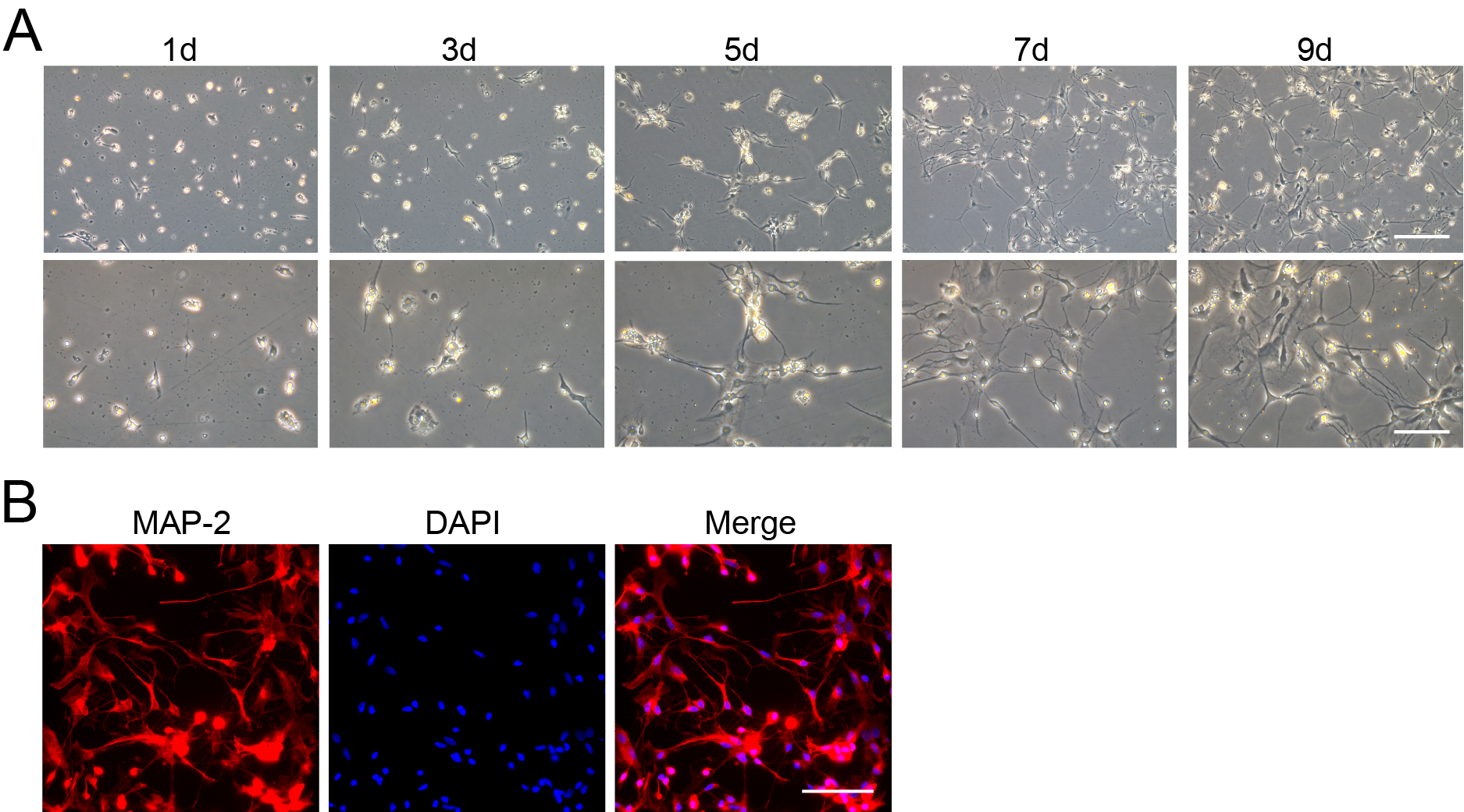

Supplement: Supplementary Figure S2 — Primary cultures of cortical neurons. (A) The number and morphology of primary neurons at different time points. Scale bars = 200 (upper panels) and 100 μm (lower panels). (B) Neuronal purity as determined by immunofluorescence. Scale bars = 100 μm. [file Image_2.TIF]

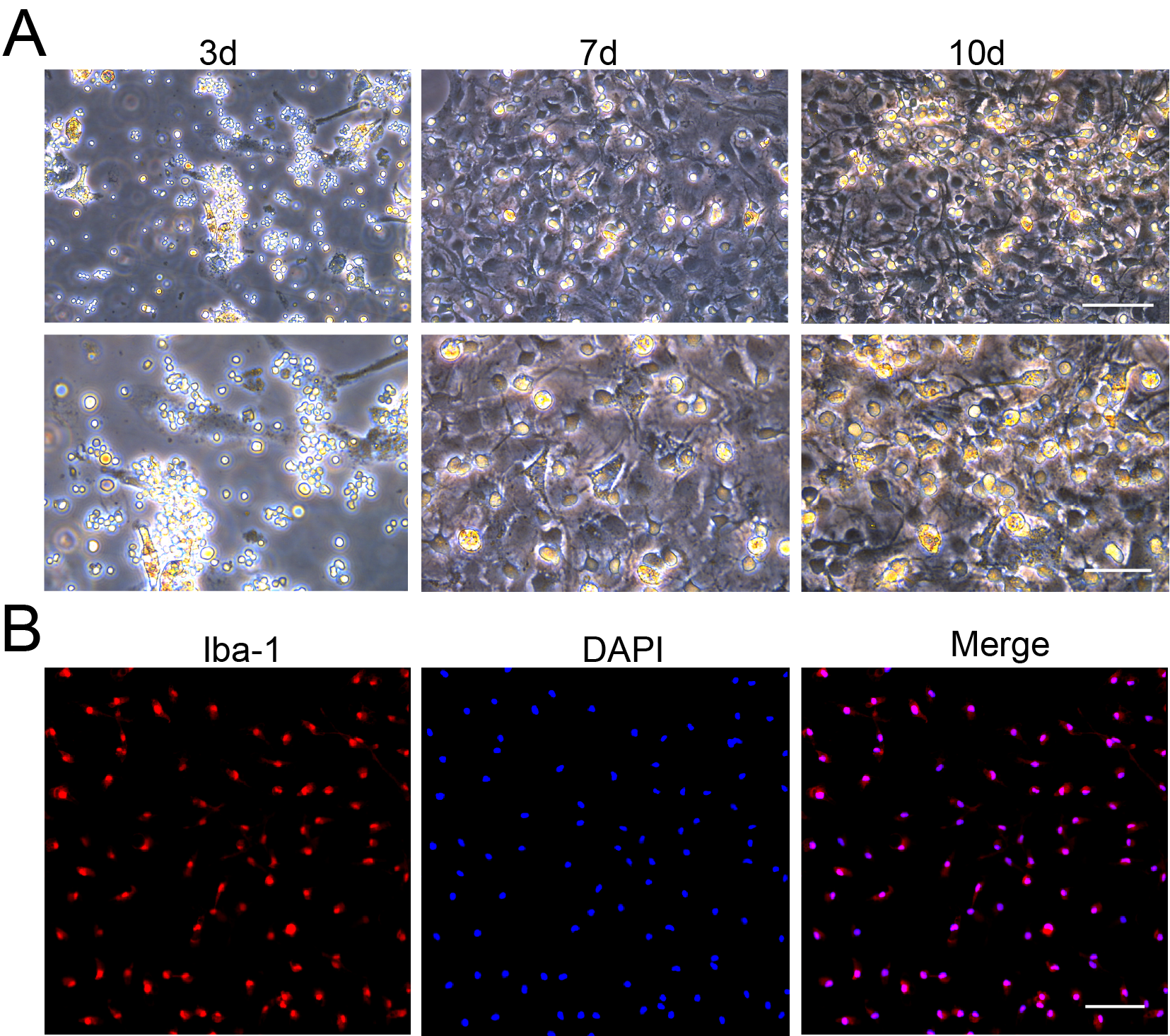

Supplement: Supplementary Figure S3 — Primary cultures of cortical microglia. (A) The number and morphology of primary microglia at different time points. Scale bars = 100 (upper panels) and 50 μm (lower panels). (B) Microglial purity as determined by immunofluorescence. Scale bars = 50 μm. [file Image_3.TIF]
